# Supplementary material for: A quantitative theory of gamma synchronization in macaque V1
Source: eLife. 2017 Aug 31;6:e26642. doi: 10.7554/eLife.26642 (PMC5779232; doi:10.7554/eLife.26642)
Supplement: Transparent reporting form [file elife-26642-transrepform.docx]

***eLife’s* transparent reporting form**

We encourage authors to provide detailed information *within their submission* to facilitate the interpretation and replication of experiments. If you have any questions, please contact us: [editorial@elifesciences.org](mailto:editorial@elifesciences.org).

**Sample-size estimation**

- You should state whether an appropriate sample size was computed when the study was being designed
- You should state the statistical method of sample size computation and any required assumptions
- If no explicit power analysis was used, you should describe how you decided what sample (replicate) size (number) to use

Please outline where this information can be found within the submission (e.g., page numbers or figure legends), or explain why this information doesn’t apply to your submission:

*In our monkey experiment, we did not know beforehand how strong effects would be (e.g. depends strongly on SNR). Sample size further depends strongly on how long the monkey wants to work (using positive rewards only).*

**Replicates**

- You should report how often each experiment was performed
- You should include a definition of biological versus technical replication
- The data obtained should be provided and sufficient information should be provided to indicate the number of independent biological and/or technical replicates
- If you encountered any outliers, you should describe how these were handled
- Criteria for exclusion/inclusion of data should be clearly stated
- High-throughput sequence data should be uploaded before submission, with a private link for reviewers provided (these are available from both GEO and ArrayExpress)

Please outline where this information can be found within the submission (e.g., page numbers or figure legends), or explain why this information doesn’t apply to your submission:

The number of subjects, number of sessions per subject, trial number and number of recording electrode contacts can be found in Supplementary materials (section Recording techniques, Line 12). Biological replications, defined here as recording distinct biological samples, correspond to different subjects (Monkeys), sessions (due to acute recording probes had different positions in V1) and electrode contacts situated at different cortical depths. Technical replications, defined here repeated measures of the same biological sample, correspond to trial number.

**Statistical reporting**

- Statistical analysis methods should be described and justified
- Raw data should be presented in figures whenever informative to do so (typically when N per group is less than 10)
- For each experiment, you should identify the statistical tests used, exact values of N, definitions of center, methods of multiple test correction, and dispersion and precision measures (e.g., mean, median, SD, SEM, confidence intervals; and, for the major substantive results, a measure of effect size (e.g., Pearson's r, Cohen's d)
- Report exact p-values wherever possible alongside the summary statistics and 95% confidence intervals. These should be reported for all key questions and not only when the p-value is less than 0.05.

We report the key statistical tests in the main manuscript methods and additional information are presented in the Suppl. Materials (section *Evaluation of prediction accuracy of analytical model*, Line 638)

We included Figure 2 and Figure 3 to show the main effects in a raw data form as much as possible.

For each statistical test, we report the name, the p-value, the number and the effect size.

All our p-values are substantially lower than 0.05 and effect sizes are considerable/strong. For all analysis and tests we report results for the two monkeys separately.

Please outline where this information can be found within the submission (e.g., page numbers or figure legends), or explain why this information doesn’t apply to your submission:

(For large datasets, or papers with a very large number of statistical tests, you may upload a single table file with tests, Ns, etc., with reference to page numbers in the manuscript.)

**Additional data files (“source data”)**

- We encourage you to upload relevant additional data files, such as numerical data that are represented as a graph in a figure, or as a summary table
- Where provided, these should be in the most useful format, and they can be uploaded as “Source data” files linked to a main figure or table
- Include model definition files including the full list of parameters used
- Include code used for data analysis (e.g., R, MatLab)
- Avoid stating that data files are “available upon request”

Please indicate the figures or tables for which source data files have been provided:

-
